# Effects of low-intensity pulsed electromagnetic fields on bone microarchitecture, mechanical strength and bone turnover in type 2 diabetic db/db mice

**DOI:** 10.1038/s41598-017-11090-7

**Published:** 2017-09-07

**Authors:** Jianjun Li, Zhaobin Zeng, Yantao Zhao, Da Jing, Chuhua Tang, Yin Ding, Xue Feng

**Affiliations:** 1 State Key Laboratory of Military Stomatology, Department of Orthodontics, School of Stomatology, Fourth Military Medical University, Xi’an, Shaanxi 710032 P.R. China; 2 Department of Stomatology, the 306th Hospital of PLA, Beijing, 100037 P.R. China; 3 Department of Stomatology, General Hospital of Shenyang Military Area Command, Shenyang, Liaoning 110015 P.R. China; 4 Beijing Engineering Research Center of Orthopedic Implants, the First Affiliated Hospital of PLA General Hospital, Beijing, 100048 P.R. China; 5 Department of Biomedical Engineering, Fourth Military Medical University, Xi’an, Shaanxi 710032 P.R. China

## Abstract

Type 2 diabetic patients have impaired bone quality, leading to increased fracture risk. Substantial evidence demonstrates that pulsed electromagnetic fields (PEMF) could resist osteopenia/osteoporosis induced by estrogen deficiency and disuse. However, the effects of PEMF on osteopenia/osteoporosis associated with diabetes, especially for more prevalent type 2 diabetes, remain poorly understood. We herein investigated the skeletal effects and mechanisms of PEMF (15 Hz, 20 Gs) on leptin receptor-deficient db/db mice with typical type 2 diabetic symptoms. Our µCT results showed that 12-week PEMF exposure significantly improved both cancellous and cortical bone microarchitecture in db/db mice. Three-point bending and biomechanical indentation testing demonstrated that PEMF improved whole-bone structural properties and tissue-level material properties in db/db mice. PEMF significantly promoted bone formation in db/db mice evidenced by increased serum osteocalcin and bone mineral apposition rate, whereas PEMF exerted no observable alteration in bone resorption. Real-time PCR showed that PEMF upregulated tibial gene expression of osteoblastogenesis-related of canonical Wnt/β-catenin signaling but not osteoclastogenesis-related RANKL-RANK signaling in db/db mice. Our findings demonstrate that PEMF improved bone quantity and quality with obvious anabolic activities in db/db mice, and imply that PEMF might become a clinically applicable treatment modality for improving bone quality in type 2 diabetic patients.

## Introduction

Diabetes mellitus (DM), as an emerging epidemic in which the organism either does not produce enough or respond to insulin, afflicts approximately 350 million people worldwide^1^. Substantial evidence suggests that DM is capable of causing various musculoskeletal abnormalities, such as imbalanced bone metabolism, poor bone healing and regeneration, and increased risk of bone fractures^2, 3^. In patients with type 1 DM (insulin-dependent DM), significant decrease in bone formation was observed, leading to reduced bone mass and increased risk of osteoporosis^4, 5^. Moreover, spontaneous and pharmacologically induced type 1 DM animals also display significant decreases in bone formation, bone mass and skeletal biomechanical strength^6–9^. Type 2 DM (noninsulin-dependent DM) patients also exhibit impaired bone microarchitecture and skeletal quality, leading to the increase in bone fragility^4, 10^. The issues pertaining to the diabetic bone complications are becoming a clinical challenge due to the sharply increased diabetic population, which often cause more pains and increased risk of fractures for DM patients. Thus, it holds great clinical significance for developing effective approaches for the prevention and treatment of diabetic osteopenia/osteoporosis.

In the past four decades, accumulating evidence suggests that treatment of pulsed electromagnetic fields (PEMF) is able to produce satisfying effects on several bone diseases, such as fresh and nonunion fractures^11, 12^, osteoarthritis^13, 14^ and bone defects^15^. Substantial studies have also shown that PEMF stimulation exerted significantly preventive effects against deterioration of bone quantity and quality in osteoporotic animals induced by ovariectomy (OVX) or hindlimb unloading^16–20^. Furthermore, several clinical trials have also revealed that PEMF are capable of significantly increasing bone mineral density and promoting osteogenesis^21–23^. On the basis of the animal and clinical findings, numerous *in vitro* studies also suggest that PEMF stimulation significantly promoted osteoblast proliferation and mineralization^24–26^ and inhibited osteoclast maturation and function^27, 28^. Considering the high cost or adverse side effects of current available anti-osteoporosis drugs (*e*.*g*., hormones and bisphosphonates) and nutrients (*e*.*g*., calcium and vitamin D)^29–33^, PEMF treatment might become a more promising alternative regimen for resisting osteoporosis due to its economic, safe and noninvasive nature. In spite of these positive experimental and clinical findings of PEMF, little is understood about the potential effects of PEMF exposure on osteopenia/osteoporosis associated with DM, especially for more prevalent type 2 DM.

Leptin is a circulating protein specifically secreted by the adipocyte, which plays a key role in regulating appetite and energy expenditure. Leptin works as a satiety factor by binding specific receptors encoded by the db gene localized in the hypothalamus when body’s energy stores are adequate. Leptin receptor-deficient db/db mice exhibit typical type 2 DM symptoms, characterized by obesity, hyperphagia, hyperglycemia and hyperinsulinemia^34^. Moreover, the leptin signaling exerts a direct anabolic action on bone. Leptin administration was able to enhance osteoblast activity and inhibited osteoclastogenesis *in vitro*, and improve bone quantity and quality in various osteopenic animals *in vivo* ^35–39^. Leptin receptor-deficient db/db mice have been shown to exhibit reduced bone formation, impaired bone architecture, and decreased bone strength^37, 40–42^. Therefore, in the present study, we hypothesize that PEMF exposure was capable of improving bone microstructure and regulating bone metabolism in type 2 DM db/db mice. To examine this hypothesis, the effects of PEMF stimulation for 12 weeks on bone quantity, quality and turnover in db/db mice were systematically evaluated via serum biochemical, bone biomechanical, µCT and histomorphometric analyses. Moreover, the potential regulatory mechanisms of bone turnover in db/db mice under the exposure of PEMF were also preliminarily elucidated.

## Materials and Methods

### Animals

Twelve-week-old, skeletally mature male diabetic db/db mice on the C57BKS background (BKS.Cg-*m* +/+ *Lepr* ^*db*^/J) and their homozygous littermate wild-type control (WT) mice purchased from the Jackson Laboratory (Bar Harbor, Maine) were used in the current study. All animals were acclimatized to the laboratory environment for one week. Blood samples of all mice were collected from the tail vein and fasting blood glucose levels were quantified using a glucometer (OneTouch SureStep Plus, Lifescan, Milpitas, CA). The db/db mice with fasting blood glucose >16.7 mmol/L was considered to be qualified diabetic models. Eighteen db/db mice were randomly divided and assigned to the db/db (db/db) and db/db with PEMF stimulation (PEMF) groups (*n* = 9), and nine WT mice were used as the blank controls (WT). All animals were housed under controlled temperature (23 ± 1 °C) and relative humidity (50~60%). The lights were switched on and off at 07:00 and 19:00, respectively. Animals were allowed ad libitum access to clean tap water and standard rodent chow. The db/db mice in the PEMF group were subjected to 2 h/day whole-body PEMF exposure. The effectiveness of this PEMF treatment duration (2 h/day) has been verified on promoting fracture/defect healing and increasing bone mass by studies from our group and others^15, 19, 43^. Mice in the PEMF group were exposed with PEMF for 12 weeks. This 12-week PEMF exposure period has been shown effective both experimentally and clinically on resisting bone loss induced by estrogen deficiency^20, 44, 45^. All mice were given two intramuscular injections of 8 mg/kg calcein (Sigma-Aldrich) on 14 and 4 days before the animals were killed, respectively. At the end of 12-week experimental period, all mice were anesthetized by intraperitoneal injection of equithesin (42.5 mg/ml chloral hydrate, 9.7 mg/ml pentobarbital sodium and 21.3 mg/ml magnesium sulfate heptahydrate) at a dose of 0.4 mL/100 g body wt. The method of abdominal aorta puncture was used to collect the arterial blood. Then, the mice were euthanized using carbon dioxide inhalation, followed by confirmation of death with cervical dislocation. The serum samples were obtained by centrifuging the blood samples and stored at −70 °C for quantification for serum markers of bone turnover. The harvested bilateral femoral samples were wrapped in saline-soaked gauze and stored at −70 °C. Left femora were used for biomechanical 3-point bending and indentation testing, and right femora were employed for µCT scanning and dynamic bone histomorphometric analysis. Left and right tibiae were harvested and stored in liquid nitrogen for qRT-PCR and western blotting analyses, respectively. All the procedures used in animal studies were approved by the Institutional Animal Care and Use Committee of the Fourth Military Medical University, and all procedures were strictly carried out in accordance with the approved guidelines.

### PEMF apparatus

**Figure 1:**
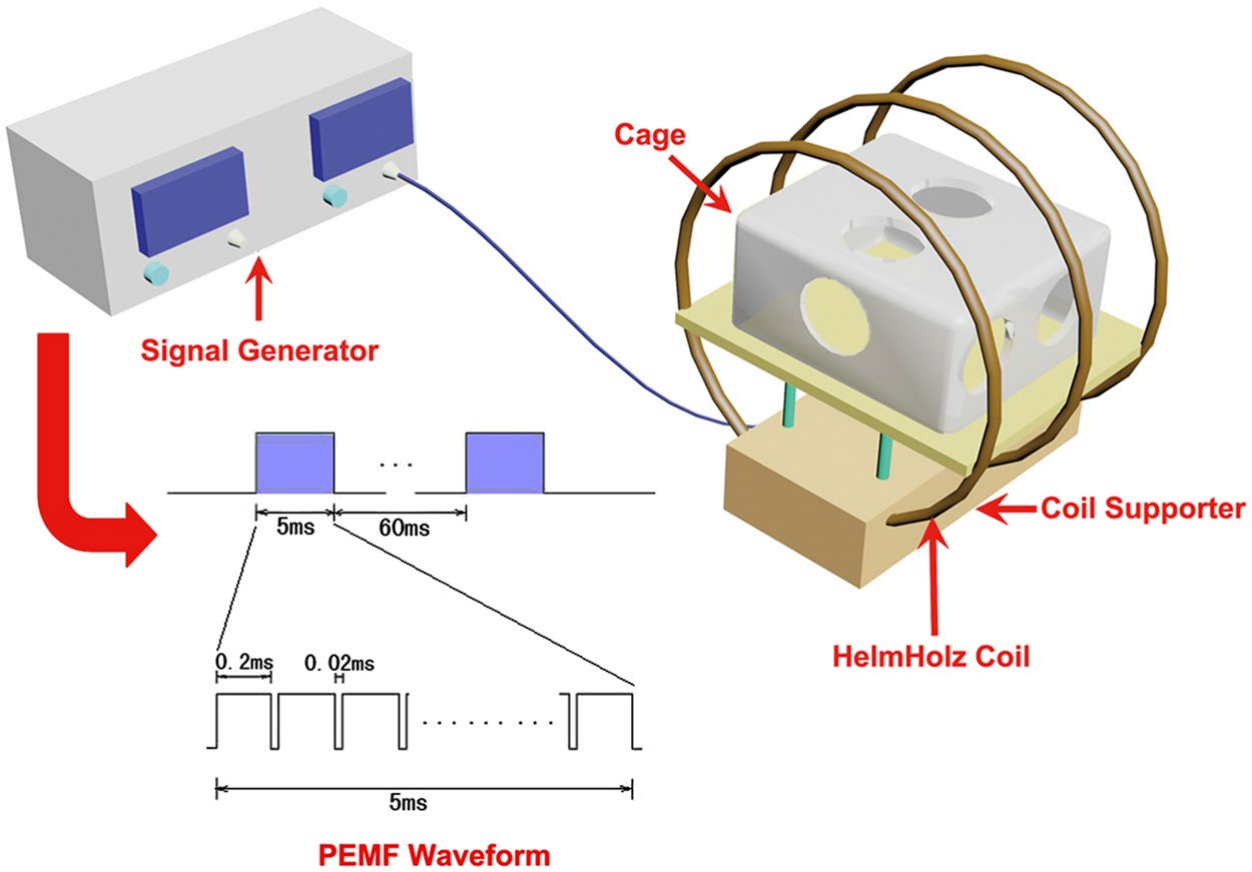
Schematic representation of the PEMF apparatus and PEMF waveform. The PEMF exposure system consisted of a signal generator and a Helmholtz coil assembly with three coils. The three coils (80-cm diameter) were placed coaxially with 30.4 cm apart from each other, and the numbers of turns of the central coil and outside coils were 266 turns and 500 turns, respectively. The bottom of the plastic mouse cage was aligned with the center of the coils to ensure that the mice were confined in the center of the electromagnetic fields. The PEMF waveform consisted of a pulsed burst (burst width, 5 ms; pulse width, 0.2 ms; pulse wait, 0.02 ms; burst wait, 60 ms; pulse rise and fall times: 0.3 μs and 2.0 μs) repeated at 15 Hz. The peak magnetic field generated at the center of the Helmholtz coils was approximately 2.0 mT.

A custom-designed electromagnetic exposure system was used in the present study to generate a PEMF waveform comprising a pulsed burst (burst width, 5 ms; pulse width, 0.2 ms; pulse wait, 0.02 ms; burst wait, 60 ms; pulse rise, 0.3 μs; pulse fall, 2.0 μs) repeated at 15 Hz (Fig. 1). This waveform has shown satisfying effects on resisting osteopenia in disuse and OVX animals^19, 20^. The electromagnetic exposure system consisted of a signal generator and a Helmholtz coil assembly (Fig. 1). The three coils (80-cm diameter) were placed coaxially with 30.4 cm apart from each other, and the numbers of turns of the central coil and outside coils were 266 turns and 500 turns, respectively. The assembly of three coils has been proved to exhibit higher magnetic field uniformity than traditional assembly of two coils^20^. The bottom of the plastic mouse cage was aligned with the center of the coils to ensure that the mice were confined in the center of the electromagnetic fields. In order to calculate the current in the coils, a resistor of 2 Ω was placed in series with the coils, and the voltage drop across the resistor was observed with an oscilloscope (Agilent Technologies, Santa Clara, CA). Based on the measured current value, the peak magnetic field of the coils was calculated to be approximately 2.0 mT. The accuracy for the peak magnetic field measurement was confirmed by the Gaussmeter measurement (Model 455 DSP, Lake Shore Cryotronics, Westerville, OH). The measured background electromagnetic field was 50 ± 2 µT. To determine the induced electric field within the coils, a custom-designed electrical potential detecting circular coil (5 cm coil diameter, 1 mm coil diameter, 20 turns) was placed in the midcenter of the Helmholtz coils with the coil parallel to the Helmholtz coils. The current detecting coil was connected with the oscilloscope, and the induced peak electrical field was determined to be approximately 2 mV/cm.

### Quantification for serum markers of bone turnover

Osteocalcin (OCN) is specifically secreted by osteoblasts, and serum OCN is regarded as a good indicator for bone formation^46^. Tartrate-resistant acid phosphatase 5b (TRACP5b) is specifically produced by osteoclasts, and serum TRACP5b has proved to be a good marker for indicating osteoclast activity and bone resorption rate^47^. Serum OCN and TRACP5b concentrations (*n* = 9 for each group) of all mice in the current study were quantified using commercial enzyme-linked immunosorbent assay (ELISA) kits (CUSABIO Biotech Co., Wuhan, China) according to the manufacturer’s instructions.

### Trabecular and cortical bone microstructure analysis

Bone microarchitecture of right femora of all mice were evaluated using a high-resolution µCT scanning system (GE healthcare, Madison, WI). Femoral bone samples were placed in a 10-mm-diameter tube perpendicularly to the scanning axis. The µCT scanning parameters chosen for this study include: voltage 80 kV, current 80 μA, exposure time 2.96 s, total rotation angle 210°, and rotation angle of increment 0.4°. After scanning, 2-dimensional image sequences were reconstructed to the 3-dimensional image with an isotropic voxel size of 15.9 μm. To analyze and quantify femoral trabecular bone microarchitecture, a volume of interest (VOI) with 1.0-mm height started at a distance of 0.5 mm from the lowest end of the growth plate of the distal femur and extended to the proximal end with a distance of 1.0 mm was selected, which only contained the second spongiosa. Another VOI with 2.0-mm height was selected to analyze the cortical bone structure of the femoral mid-diaphysis. All 3D image manipulations and analyses were performed using the system software (MicroView, v.2.1, GE healthcare). The following trabecular and cortical bone architectural parameters were determined, including trabecular bone volume per tissue volume (BV/TV), trabecular number (Tb.N), trabecular thickness (Tb.Th), trabecular separation (Tb.Sp), cortical thickness (Ct.Th) and cortical area (Ct.Ar).

### Bone histomorphometric analysis

After µCT scanning, femoral samples were dehydrated in 80% ethanol for 2 days and embedded in methylmethacrylate (MMA). Then, a diamond saw microtome (Leica 2500E, Leica SpA, Milan, Italy) was used to longitudinally section the femoral samples along the axial plane to approximately 50 μm thickness. The calcein double-labeling sections were imaged using a fluorescence microscope (LEICA DM LA, Leica Microsystems, Heidelberg, Germany). The following dynamic endocortical bone histomorphometric parameters were analyzed and quantified (*n* = 9 for each group), including mineral apposition rate (MAR, µm/day, the average distance between the two calcein labels divided by the 10-day labeling intervals) and bone formation rate per bone surface (BFR/BS, µm^3^/µm^2^/day, calculated as MAR*MS/BS, MS/BS was calculated as half of single-labeled surface per bone surface plus double-labeled surface per bone surface). For bone histomorphometric analysis, one section was measured for each sample, and all sections were coded and analyzed ‘blind’ by one independent observer.

### Biomechanical 3-point bending testing

To evaluate the whole-body biomechanical structural properties, 3-point bending testing was performed on the left femora of all mice (*n* = 9 for each group) using a servohydraulic materials testing machine (Bose ElectroForce 3220, Bose Corp, Eden Prairie, MN). Before mechanical testing, all frozen bone samples were thawed in physiological saline solution for 1 h. The whole femoral sample with its physiological curvature facing up was immobilized on a supporter with two fixed loading points with 8-mm distance apart from each other. A static 0.5 N preload was applied on the surface of the femur mid-shaft to immobilize the sample by controlling the motion of the upper loading axis, which was perpendicularly aligned to the long axis of the femur and located at the midpoint between the two lower loading points. Then, loading was applied by controlling the motion of the upper loading axis until an ultimate fracture occurred at a constant 0.02 mm/sec displacement rate. Force versus displacement data were collected, and maximum load (N, maximum tensile load that the femur can sustain before failure) and stiffness (N/mm, slope of the linear part of the curve representing elastic deformation) were quantified according to the load-deformation curve.

### Biomechanical indentation examination

Biomechanical indentation testing was performed using a commercial indentation system (Agilent G200, Agilent Technologies Inc., Chandler, AZ, USA) equipped with a Berkovich diamond tip. A low-speed saw (Leica 2500E, Leica SpA, Milan, Italy) was used to transversely cut the femora, and the distal femora were dehydrated in graded ethanol and embedded in MMA. The bone surfaces were polished with silicon carbide abrasive paper of progressively finer grit (800, 1000 and 1200) under 2-min water irrigation. Samples were then rehydrated in saline solution for 24 h. The indentation tip contact area function of the indentation machine was calibrated using the fused silica before mechanical testing on bone^48^. The biomechanical indentation was conducted under a load-control mode. The indenter tip was loaded into the bone sample to reach a maximum depth for 4 µm at a constant strain rate with 0.05 sec^−1^, followed by 10-sec holding at the peak load for minimizing the viscoelastic behavior and creep of bone tissue. Then, the unloading was applied to 10% of the peak load at the maximum loading rate of each indentation point, followed by a 60-sec holding for calculating the thermal drift. The indenter was finally removed from the bone surface. The force-displacement curve was automatically collected. The parameters reflecting intrinsic material properties, including elastic modulus and contact hardness were calculated as previously described^48^. For each bone sample, indentations were conducted in a grid of 3 × 3 points with 10 μm spacing (9 indentations were made in each bone sample). The average values for the elastic modulus and contract hardness of trabecular bone tissue were calculated for each bone sample. A total of 243 indents were included for the overall 27 mice in the three groups. All indentation examinations were performed by a technician blinded to the samples in the three groups.

### RNA extraction and qRT-PCR

Left tibiae were dissected from all mice after scarification (*n* = 9 for each group). After removal of soft tissues and muscles on ice, tibial mid-diaphysis with 0.5-cm length was extracted using the Leica 2500E low-speed saw, and then repeatedly flushed with 0.1 M ice-cold PBS to remove bone marrow. Samples were snap-frozen in liquid nitrogen to make them breakable. To collect the RNA samples, tibial mid-diaphysis samples were crushed into powder using the pestle in a mortar containing liquid nitrogen and mixed with the monophasic solution of phenol and guanidine thiocyanate. Total RNA was extracted using the guanidinium isothiocyanate-alcohol phenyl-chloroform method according to the manufacturer’s protocol (Invitrogen, Carlsbad, CA). Then, cDNA was synthesized from RNA using SuperScript III reverse transcriptase. PCR was performed using the SYBR Green PCR Master Mix (Applied Biosystems, Foster City, CA) on ABI 7300 Real-Time PCR system. The primers used in the current study are shown in Table 1. Each sample was run for Real-Time PCR analysis in triplicate in a 96-well plate, and the relative quantity of mRNA (normalized to the house-keeping gene β-actin content) was calculated using 2^−ΔΔCt^ relative quantification methods. All qRT-PCR reactions for each tibial sample were performed three times.

**Table 1:** The sequence of primers used in the current study for Real-time PCR analysis.

Genes	Forward primer (5′-3′)	Reverse primer (5′-3′)
Osteocalcin	GTGTGAGCTTAACCCTGC	ACAGGGAGGATCAAGTCC
Runx2	TGCACCTACCAGCCTCACCATAC	GACAGCGACTTCATTCGACTTCC
BMP2	AGAAAAGCAACAGAAGCC	GACCGCAGTCCGTCTAAG
OPG	ACCAAAGTGAATGCCGAGAGAG	ACGCTGCTTTCACAGAGGTC
RANKL	GGGGAGGCAACTGTCACCTT	TAGTCTGTAGGTACGCTTCC
RANK	CACGGTGGATTCTGAGGGCT	GGGGAGGCAACTGTCACCTT
Wnt3a	CTCCTCTGCAGCCTGAAGC	GTGGACGGTGGTGCAGTT
Lrp6	CAGCACCACAGGCCACCAA	TCGAGACATTCCTGGAAGAG
β-catenin	GGAAAGCAAGCTCATCATTCT	AGTGCCTGCATCCCACCA
β-actin	GCCAACACAGTGCTGTCT	AGGAGCAATGATCTTGATCTT

### Bone protein extraction and western blotting analysis

After animal sacrifice, soft tissues and muscles from right tibiae were immediately removed on ice. Both ends of the tibia were removed using the Leica 2500E low-speed saw to ensure that the remaining tibial mid-diaphysis with 0.8-cm length was used for western blotting analysis. After removal of bone marrow, tibial samples were pulverized using the pestle in a mortar containing liquid nitrogen. The protein in tibial samples was extracted using a protein extraction kit specialized for bone tissues (BestBio, Shanghai, China). The protein extracts (40 µg per sample) were separated by 10% Tris-glycine SDS-PAGE and then transferred onto PVDF membranes (EMD Millipore, Billerica, MA) after mixed with 5× loading buffer. The PVDF membranes were blocked in TBST (Tris Buffer Saline, 0.5% Tween-20) containing 5% BSA for 1 h and incubated with primary antibodies to Wnt3a (Bioss, Beijing, China), Lrp6 (Bioss, Beijing, China), β-catenin (EMD Millipore, Billerica, MA) and β-tubulin (Bioworld technology, Inc., Louis Park, MN) in TBST containing 5% BSA overnight at 4 °C. β-tubulin was used as an internal control for normalization. Then, the membranes were incubated with a 1:3000 dilution of HRP-conjugated secondary antibody for 1 h at room temperature. The protein levels were assayed using an ECL chemiluminescence system (GE ImageQuant 350, GE Healthcare). Semi-quantitative analysis was carried out using the QuantityOne Software (Bio-Rad).

### Statistical analysis

All data shown in the current study were expressed as the mean ± standard deviation (S.D.). The Kolmogorov-Smirnov test was used to examine the normal distribution of all data, and the Levene’s test was employed to evaluate the homogeneity of variance. Our analysis demonstrated that each specific parameter in the three groups obeyed normal distribution and homoscedasticity. Statistical significance between every two groups was determined using one way analysis of variance (ANOVA) followed by a Bonferroni’s post-hoc multiple comparison. *P* < 0.05 was considered to be the criteria for statistical significance.

## Results

### Body weight and blood glucose levels

**Figure 2:**
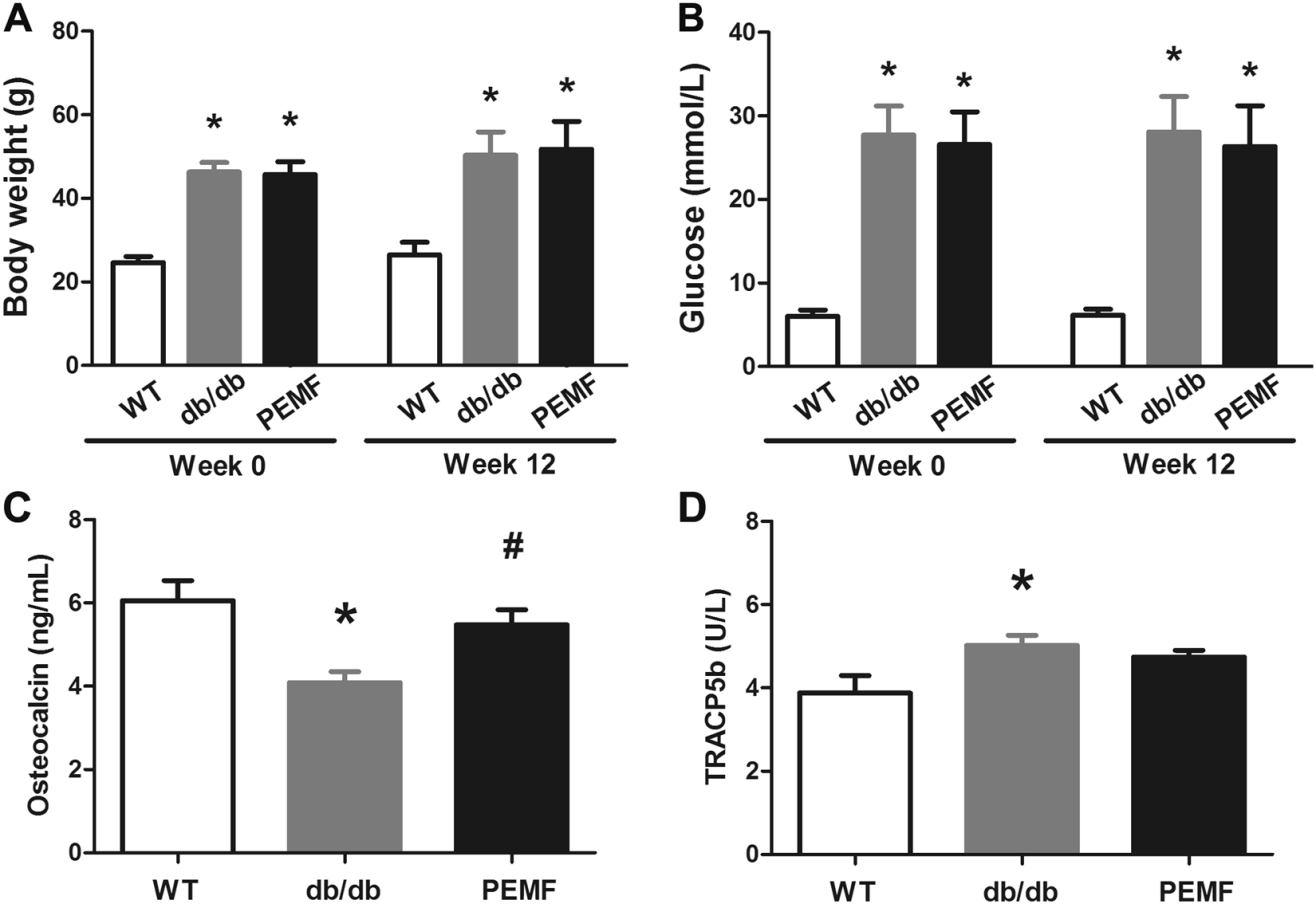
Effect of PEMF stimulation on body weight, blood glucose and serum bone turnover markers in *db/db* mice. The db/db mice showed significantly higher body weight (**A**) and blood glucose (**B**) than the WT group both before and after 12-week experiment, whereas 12-weeek PEMF exposure exhibited no apparent effect on body weight or blood glucose in db/db mice. The db/db mice also exhibited decrease in bone formation marker (**C**) osteocalcin (OCN) and increase in bone resorption marker (**D**) tartrate-resistant acid phosphatase 5b (TRACP5b). PEMF promoted serum OCN secretion, whereas PEMF displayed no observable effect on serum TRACP5b expression. WT, the wild-type mice group; db/db, the db/db mice group; PEMF, the db/db mice with PEMF stimulation group. Values are all expressed as mean ± S.D. (*n* = 9). ^*^Significant difference from the WT group with *P* < 0.05; ^#^Significant difference from the db/db group with *P* < 0.05.

The body weight and blood glucose levels of mice before and after 12-week PEMF exposure are shown in Fig. 2A and B. Body weight and blood glucose in the db/db group and PEMF group were significantly higher than those in the WT group before PEMF stimulation (*P* < 0.01). After 12-week PEMF exposure, the db/db mice exhibited significantly higher body weight and blood glucose levels than the WT mice (*P* < 0.01), whereas no obvious difference was observed in body weight and blood glucose between the db/db group and PEMF group (*P* = 1.000 and *P* = 0.999, respectively).

### Serum biochemical analysis

The serum biomarkers for bone formation and bone resorption are depicted in Fig. 2C and D. Serum OCN, a critical biomarker for bone formation, was significantly lower in the db/db group than that in the WT group (*P* = 0.003). The db/db mice subjected to 12-week PEMF exposure showed significantly increased serum OCN concentration as compared with the db/db group (*P* = 0.039, +34.1%). The db/db group also displayed significant elevation in serum TRACP5b levels as compared with the WT group (*P* < 0.001); nonetheless PEMF stimulation exhibited no significant alteration for the secretion of serum TRACP5b in db/db mice (*P* = 0.362).

### µCT analysis

**Figure 3:**
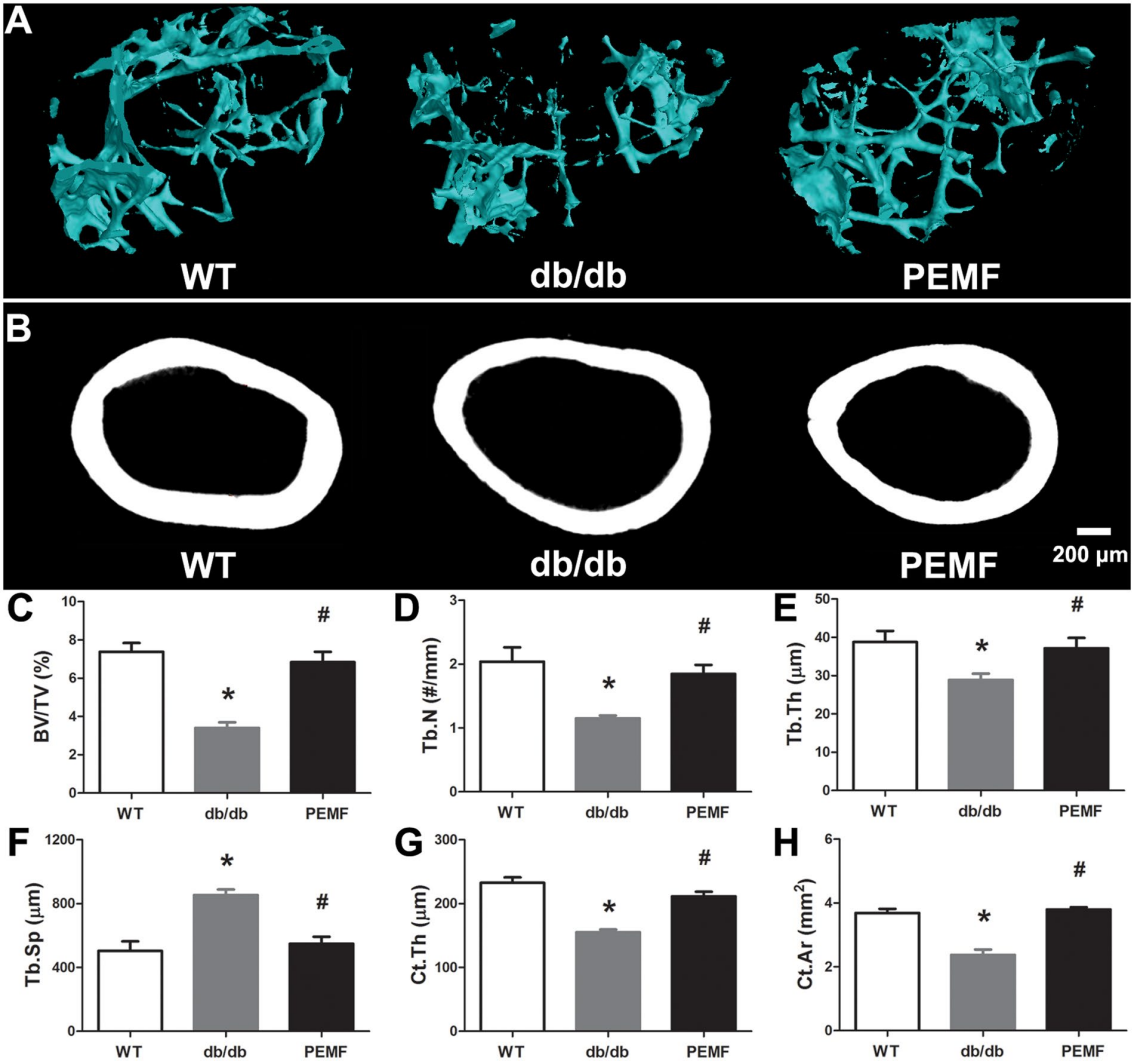
Effect of PEMF exposure on trabecular bone microarchitecture in distal femora and cortical bone thickness in femoral mid-diaphysis of *db/db* mice via µCT analysis. A volume of interest (VOI) with 1.0-mm height started at a distance of 0.5 mm from the lowest end of the growth plate of the distal femur and extended to the proximal end with a distance of 1.0 mm was selected for quantifying cancellous bone microstructure, which only contained the second spongiosa. Another VOI with 2.0-mm height was selected to analyze the cortical bone structure of the femoral mid-diaphysis. (**A~B**) Representative µCT images showing obvious deterioration of both cancellous and cortical bone microstructure in db/db mice were ameliorated after 12-week exposure to PEMF. (**C~H**) Statistical quantification of trabecular and cortical bone microarchitecture parameters, including (**C**) bone volume per tissue volume (BV/TV), (**D**) trabecular number (Tb.N), (**E**) trabecular thickness (Tb.Th), (**F**) trabecular separation (Tb.Sp), (**G**) cortical thickness (Ct.Th) and (**H**) cortical area (Ct.Th). PEMF stimulation for 12 weeks significantly improved cancellous and cortical bone microarchitecture in db/db mice. WT, the wild-type mice group; db/db, the db/db mice group; PEMF, the db/db mice with PEMF stimulation group. Values are all expressed as mean ± S.D. (*n* = 9). ^*^Significant difference from the WT group with *P* < 0.05; ^#^Significant difference from the db/db group with *P* < 0.05.

Representative µCT images (Fig. 3A and B) demonstrate obvious deterioration of cancellous bone microstructure in db/db mice as compared with the WT mice. Moreover, the db/db mice also exhibited expanded bone marrow cavity and decreased cortical bone thickness in comparison with the WT mice. However, the trabecular and cortical bone microarchitecture in db/db mice was significantly improved after exposure to PEMF for 12 weeks. Statistical quantifications (Fig. 3C~H) reveal that the db/db mice exhibited significantly compromised BV/TV (*P* < 0.001), Tb.N (*P* < 0.001), Tb.Th (*P* = 0.03) and increased Tb.Sp (*P* < 0.001) in comparison with the WT mice. Furthermore, the cortical bone parameters in db/db mice (Ct.Th and Ct.Ar) were significantly lower than those in the wild type mice (*P* < 0.001). PEMF exposure for 12 weeks significantly improved trabecular bone microarchitecture in db/db mice, as evidenced by increased BV/TV (*P* < 0.001, +100.9%), Tb.N (*P* = 0.013, +60.4%), Tb.Th (*P* = 0.044, +28.7%) and decreased Tb.Sp (*P* < 0.001, −35.6%). Moreover, PEMF stimulation also significantly increased Ct.Th (*P* < 0.001, +36.3%) and Ct.Ar (*P* < 0.001, +60.0%) in db/db mice.

### Histomorphometry

**Figure 4:**
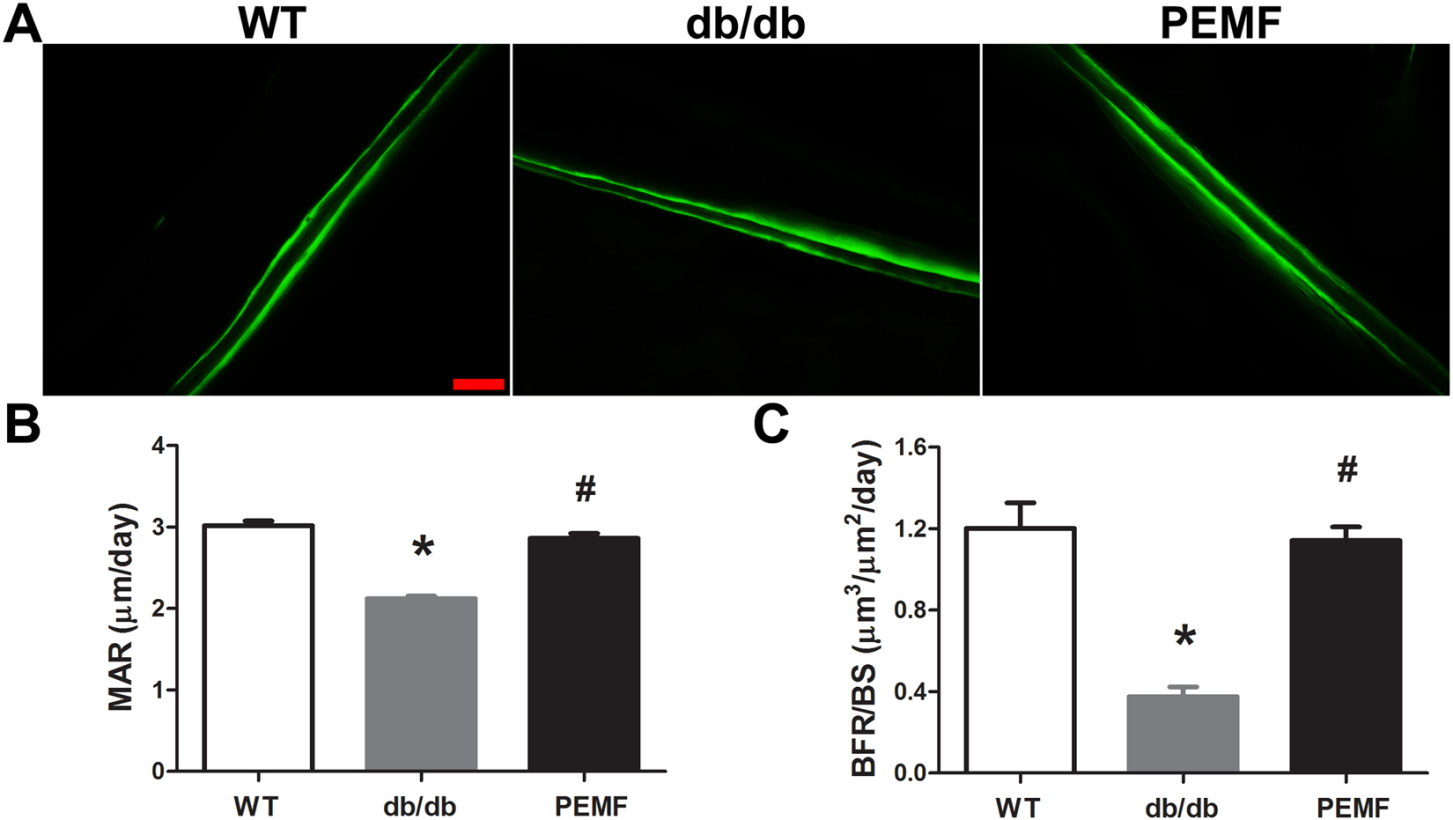
Effect of 12 weeks of PEMF stimulation on dynamic bone formation in *db/db* mice. (**A**) Representative calcein double-labeling images in femoral endocortical bone surfaces. Scale bar represents 100 µm. (**B~C**) Comparisons of dynamic histomorphometric parameters of endocortical bone between the WT, db/db and PEMF groups via double labeling with calcein, including (**B**) mineral apposition rate (MAR) and (**C**) bone formation rate per bone surface (BFR/BS). The bone formation rate in db/db mice was significantly increased after exposure to 12-week PEMF stimulation. WT, the wild-type mice group; db/db, the *db/db* mice group; PEMF, the db/db mice with PEMF stimulation group. Values are all expressed as mean ± S.D. (*n* = 9). ^*^Significant difference from the WT group with *P* < 0.05; ^#^Significant difference from the db/db group with *P* < 0.05.

Dynamic histomorphometric results via dual calcein labeling are shown in Fig. 4. Representative calcein double-labeling images on the endocortical surfaces reveal that the width of the calcein double-labeled interval in the db/db group was obviously lower than that in the WT group and the PEMF group (Fig. 4A). Statistical comparisons as shown in Fig. 4B and C demonstrate that mice in the db/db group exhibited significantly lower new bone deposition rate than the WT mice, as evidenced by decreased MAR and BFR/BS quantified by calcein double labeling analysis on the endocortical surfaces (*P* < 0.001). However, PEMF stimulation for 12 weeks significantly increased the MAR and BFR/BS levels in db/db mice (*P* < 0.001, +35.0% and +241.6%, respectively).

### Three-point bending testing

**Figure 5:**
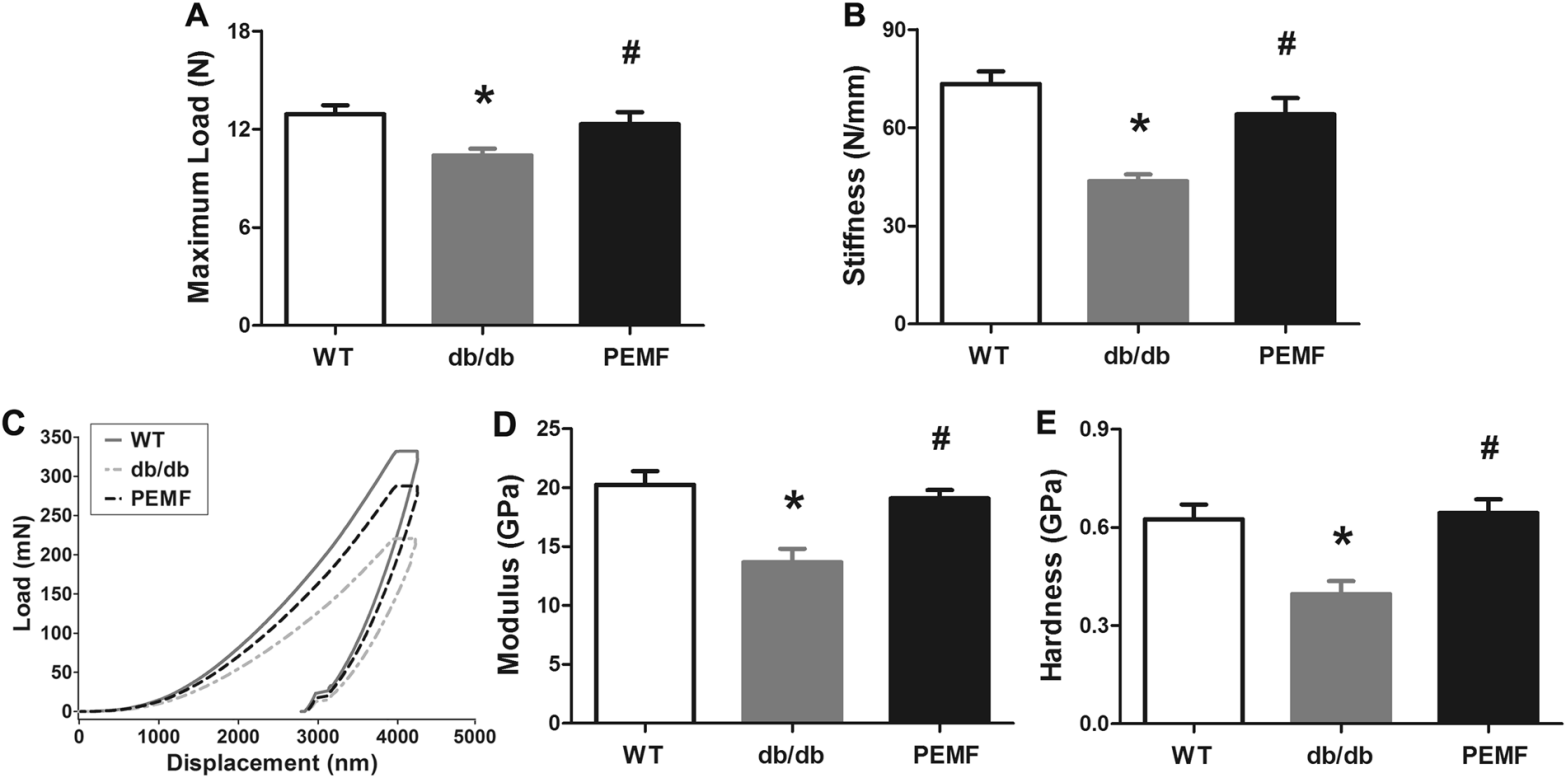
Effect of PEMF stimulation on femoral biomechanical structural and material properties in db/db mice. Three-point bending results showed that the decreases of femoral (**A**) maximum load and (**B**) stiffness in db/db mice were significantly inhibited after 12-week exposure to PEMF. (**C**) Representative load-displacement curves of biomechanical indentation testing on mouse trabecular bone in the three groups. (**D** and **E**) Statistical comparisons of cancellous bone material properties via biomechanical indentation testing in the three groups, including (**D**) elastic modulus and (**E**) hardness. Our biomechanical indentation results demonstrated that PEMF stimulation led to significant improvement of cancellous bone material properties in db/db mice. WT, the wild-type mice group; db/db, the db/db mice group; PEMF, the db/db mice with PEMF stimulation group. Values are all expressed as mean ± S.D. (*n* = 9). ^*^Significant difference from the WT group with *P* < 0.05; ^#^Significant difference from the db/db group with *P* < 0.05.

The quantification results of femoral structural properties via three-point bending test are shown in Fig. 5A and B. The structural parameters of the mouse femora quantified via three-point bending examination, including maximum load and stiffness in the db/db group were significantly lower than those in the WT group (*P* = 0.011 and *P* < 0.001, respectively). However, PEMF stimulation for 12 weeks results in significant increase in the maximum load (*P* = 0.039, +18.4%) and stiffness (*P* < 0.001, +46.6%) levels in diabetic mice as compared with the db/db group.

### Biomechanical indentation testing

The intrinsic material properties of mouse femoral trabecular bone quantified via biomechanical indentation examination are shown in Fig. 5C~E. The tissue-level modulus and hardness of mouse femoral trabecular bone in the db/db group were significantly lower than the WT group (*P* < 0.001). After 12-week PEMF exposure, mice in the PEMF group exhibited significantly improved intrinsic biomechanical material properties, including increased modulus (*P* = 0.003, +39.5%) and hardness (*P* = 0.002, +62.6%) as compared with the db/db group.

### qRT-PCR analysis

**Figure 6:**
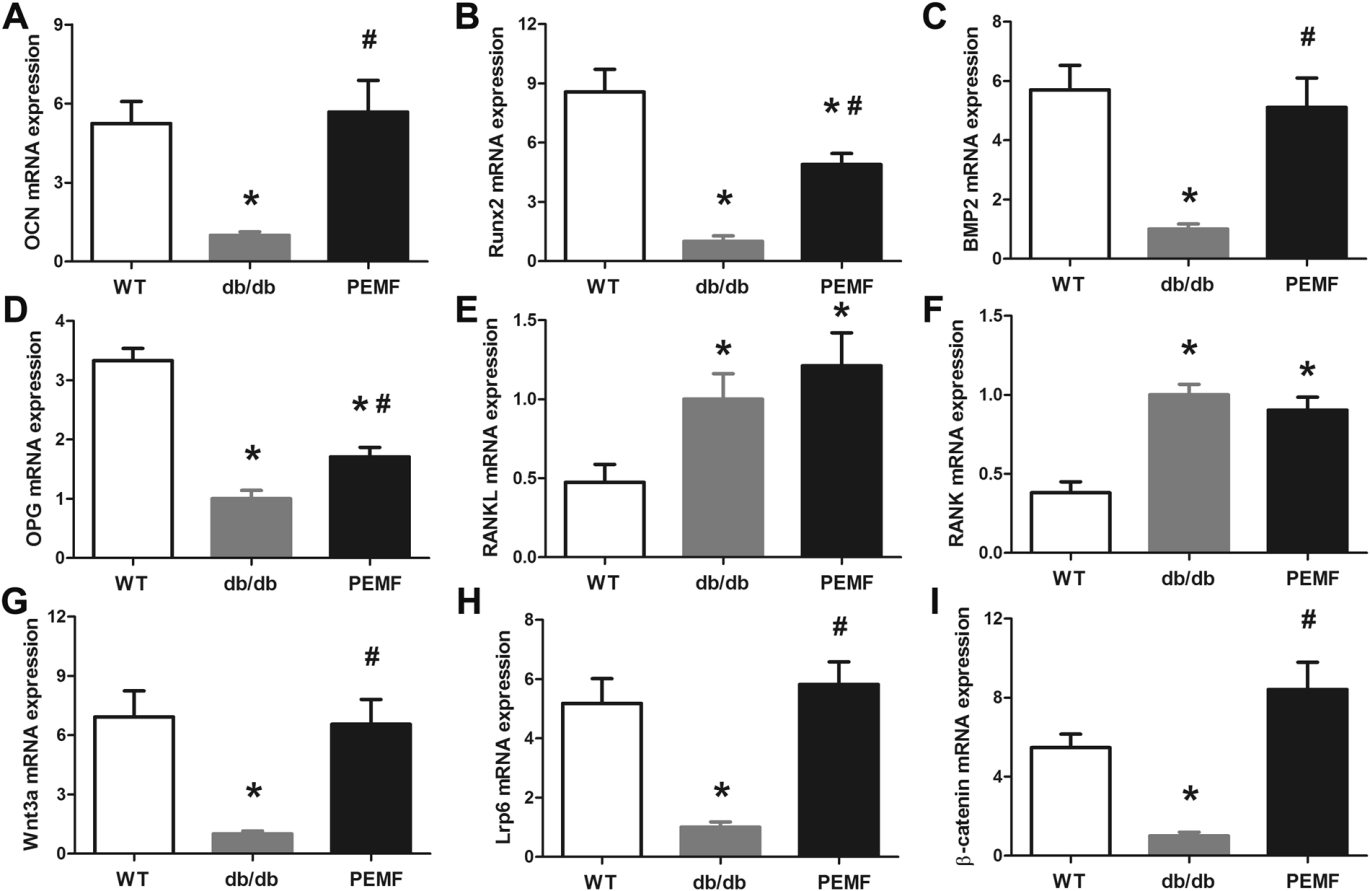
Effect of PEMF stimulation on gene expression in the mid-diaphysis of the tibia (5.0-mm length) with removal of bone marrow in db/db mice via qRT-PCR analyses, including (**A**) OCN, (**B**) Runx2, (**C**) BMP2, (**D**) OPG, (**E**) RANKL, (**F**) RANK, (**G**) Wnt3a, (**H**) LRP6, and (**I**) β-catenin mRNA expression. The left tibiae of all mice in the three groups (*n* = 9 in each group) were used for qRT-PCR analysis. Each sample was run for Real-Time PCR analysis in triplicate in a 96-well plate, and the relative quantity of mRNA (normalized to the house-keeping gene β-actin content) was calculated using 2^−ΔΔCt^ relative quantification methods. The mRNA data in the WT and PEMF groups were expressed as fold increase over levels in the db/db group. All qRT-PCR reactions for each tibial sample were performed three times. WT, the wild-type mice group; db/db, the db/db mice group; PEMF, the db/db mice with PEMF stimulation group. Values are all expressed as mean ± S.D. (*n* = 9). ^*^Significant difference from the WT group with *P* < 0.05; ^#^Significant difference from the db/db group with *P* < 0.05.

The quantification results of mouse tibial gene expression via qRT-PCR analysis are depicted in Fig. 6. The db/db group displayed significant decreases in osteogenesis-related mRNA expression in comparison with the WT group (Fig. 6A~C), as evidenced by lower OCN, Runx2 and BMP2 gene expression levels (*P* < 0.001). PEMF treatment for 12 weeks significantly promoted tibial gene expression associated with osteogenesis in db/db mice, including OCN, Runx2 and BMP2 (*P* < 0.001). We also found significantly decreased OPG mRNA expression (*P* < 0.001) and increased osteoclastogenesis-related RANKL (*P* = 0.034) and RANK (*P* < 0.001) gene expression in the db/db group as compared with the WT group (Fig. 6D~F). PEMF exposure upregulated tibial OPG gene expression in db/db mice (*P* = 0.025), whereas PEMF exerted no significant impacts on osteoclastogenesis-related RANKL or RANK mRNA expression in db/db mice (*P* = 1.000). Furthermore, mice in the db/db group also showed significant compromised tibial gene expression of the canonical Wnt signaling, including Wnt3a, Lrp6 and β-catenin (Fig. 6G~I, *P* < 0.001). Twelve-week PEMF stimulation induced significant increases of Wnt3a, Lrp6 and β-catenin gene expression in db/db mice (*P* < 0.001).

### Western blotting analysis

**Figure 7:**
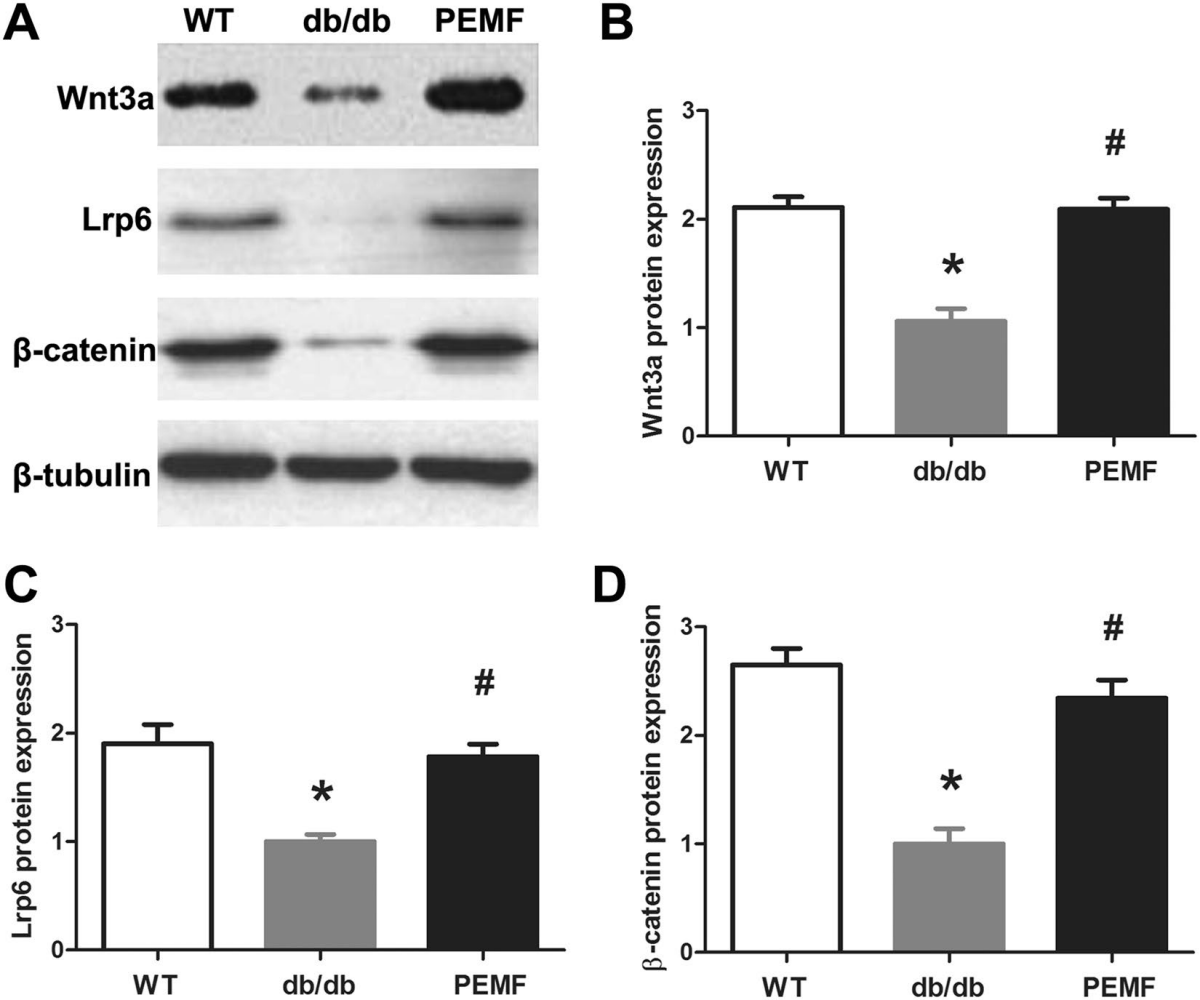
Effect of PEMF stimulation on the canonical Wnt signaling protein expression in the mid-diaphysis of the tibia (0.8-mm length) with removal of bone marrow in db/db mice. The right tibiae of 4 mice in the three groups (*n* = 4 in each group) were used for western blotting analysis. (**A**) Representative western blotting results in the Wnt3a, Lrp6, and β-catenin protein expression in the three groups. (**B~D**) Statistical comparisons of protein expression of the canonical Wnt signaling between the WT, db/db and PEMF groups via western blotting analysis, including (**B**) Wnt3a, (**C**) Lrp6, and (**D**) β-catenin. The data were normalized to the house-keeping gene β-tubulin content. The data in the WT and PEMF groups were expressed as fold increase over levels in the db/db group. WT, the wild-type mice group; db/db, the db/db mice group; PEMF, the db/db mice with PEMF stimulation group. Values are all expressed as mean ± S.D. (*n* = 4). ^*^Significant difference from the WT group with *P* < 0.05; ^#^Significant difference from the db/db group with *P* < 0.05.

The results of mouse tibial protein expression via western blotting analysis are shown in Fig. 7. Mice in the db/db group exhibited significant decrease in protein expression of the canonical Wnt signaling, including Wnt3a, Lrp6 and β-catenin (*P* < 0.05). Moreover, our western blotting results revealed that PEMF stimulation for 12 weeks significantly upregulated the canonical Wnt signaling protein expression in db/db mice, including Wnt3a, Lrp6 and β-catenin (*P* < 0.05), which kept in line with our qRT-PCR findings.

## Discussion

PEMF stimulation has proven to be able to accelerate bone fracture healing and promote osteogenesis both experimentally and clinically. The positive efficiency for resisting bone loss induced by estrogen deficient and disuse using PEMF has also been proved by substantial previous studies; nevertheless, the potential effects of PEMF on bone structure and bone remodeling in DM, especially for more prevalent type 2 DM, are still poorly understood. The insulin-resistant db/db mice lacking a functional leptin receptor has been regarded as an excellent animal model thus far for investigating type 2 DM and its relevant complications (*e*.*g*., cardiovascular disease, nephropathy and ulcers)^49–51^. Several previous findings also reveal that the db/db mice exhibit significant decrease of bone formation and deterioration of bone microstructure^37, 40–42^. In the current study, we for the first time found that PEMF exposure significantly improved cancellous and cortical bone microarchitecture, increased bone mechanical strength and enhanced bone formation in db/db mice. These findings imply that PEMF stimulation, as a safe and noninvasive method, might become a clinically applicable treatment modality for bone complication associated with type 2 DM.

According to the µCT results, we found significant decrease of BV/TV, Tb.N and Tb.Th and significant increase of Tb.Sp in db/db mice compared with the WT mice, revealing that the db/db mice exhibited deterioration of trabecular bone microstructure. These results were in line with some previous findings in db/db mice^41, 42^. Furthermore, we also for the first time observed apparent deterioration of tissue-level material properties of trabecular bone in db/db mice based on our biomechanical indentation findings, including decreased modulus and hardness. More importantly, we found significant improvement of both cancellous bone microstructure and biomechanical properties according to our µCT and indentation findings after 12 weeks of PEMF stimulation, which was consistent with previous investigations reporting the inhibitive effects of PEMF on OVX and hindlimb disuse animals^16, 19, 20^. Moreover, our µCT results also showed obviously decreased mid-shaft cortical thickness in db/db mice. Similar findings were also reported by Williams *et al*.^42^. In consistency with the µCT results, our biomechanical 3-point bending examination demonstrated that the db/db mice showed lower maximum load and stiffness than the WT mice, suggesting that leptin receptor-deficient db/db mice exhibited deteriorated cortical bone structural properties and reduced fracture toughness. After exposure to PEMF for 12 weeks, the db/db mice exhibited increased maximum load and stiffness, suggesting that PEMF exposure was capable of improving cortical bone microstructure and skeletal mechanical integrity. Although obvious skeletal action was found after PEMF stimulation, no observable diabetic phenotype in the db/db mice was altered by PEMF, including body weight and blood glucose. Thus, these results indicate that PEMF preserved bone mass in db/db mice independent of body weight and blood glucose. Together, our results clearly demonstrate that PEMF stimulation exhibited the capacity of improving microstructure and mechanical performance both in cortical bone and in trabecular bone in type 2 diabetic db/db mice, which may have important clinical implication for the improvement of bone quality in type 2 DM patients.

Growing evidence has substantiated that bone formation is impaired in both spontaneous and streptozotocin-induced type 1 DM animal models^6, 7, 52, 53^. However, the bone remodeling characteristics in db/db mice with a typical symptom of type 2 DM remain poorly elucidated. Both previous *in vivo* and *in vitro* studies have substantiated that leptin acts as a critical skeletal anabolic factor^35–39^. As expected, our serum biochemical analysis shows that leptin receptor-deficient db/db mice exhibited lower serum OCN than the WT mice, revealing decreased bone formation in db/db mice. These findings were in accordance with previously reported results^37, 42^. The compromised bone formation in db/db mice was confirmed by lower MAR and BFR/BS quantified by dynamic histomorphometry and down-regulated skeletal OCN, Runx2 and BMP2 gene expression. Interestingly, we found PEMF-induced significant increase in serum OCN, cortical bone BFR/BS and skeletal OCN, Runx2 and BMP2 gene expression, revealing the obvious anabolic action of PEMF in db/db mice. Furthermore, our serum biochemical analysis also demonstrates that the db/db mice showed significant increase in TRACP5b concentration compared with the WT mice, suggesting that the db/db mice exhibited higher bone resorption rate than the WT mice. However, 12 weeks of PEMF exposure shows no observable effect on TRACP5b levels in the db/db mice. These findings were also consistent with previous studies reporting that PEMF primarily exerted stimulating bone-forming effect rather than resisting bone-resorbing action in OVX and hindlimb disuse animals^19, 54^. Thus, our results suggest that the anti-osteopenic effects of PEMF stimulation in type 2 diabetic db/db mice was primarily associated with its obviously anabolic rather than anti-catabolic action.

Canonical Wnt/β-catenin signaling plays essential roles in regulating osteoblast activity and bone-forming function, including acceleration of preosteoblast replication, stimulation of osteoblast maturation and mineralization, and inhibition of osteoblast apoptosis^55, 56^. Moreover, canonical Wnt/β-catenin signaling is also implicated in some critical osteogenesis-related gene expression, such as Runx2 and BMP2^54–58^. Canonical Wnt signaling modulates osteoblast functions by inducing intracellular β-catenin accumulation through the binding of extracellular Wnts to Frizzled and Lrp5/6 co-receptors on plasma membrane. In our current study, we found significant decreases of gene and protein expression of Wnt3a, Lrp6 and β-catenin in the db/db mice compared with those in the WT mice, indicating that the function of canonical Wnt/β-catenin signaling was impaired. These findings were in line with our serum biochemical and dynamic histomorphometric results, and confirm that the type 2 diabetic db/db mice exhibited compromised osteoblast activity and bone formation rate. However, PEMF stimulation for 12 weeks significantly upregulated Wnt3a, Lrp6 and β-catenin gene and protein expression in the db/db mice, implying the activation of canonical Wnt signaling in the presence of exogenous PEMF signals. Several previous *in vitro* studies have shown that exogenous electromagnetic stimulation was able to trigger the transient uptake of intracellular calcium levels in osteoblasts^26, 59^. The calcium transients can induce more dephosphorylation of Ca^2+^-sensitive NFAT (nuclear factor of activated T-cells) transcription factors, and the dephosphorylated NFAT can translocate from the cytoplasm to the nucleus to initiate osteogenesis-related gene transcription (*e*.*g*., Runx2, BMP2, Wnt3a)^60, 61^. This may be one of the possible mechanisms by which PEMF upregulated osteogenesis-related gene and protein expression observed in the current study, which remains to be systematically verified in our succeeding studies. Furthermore, we also found that the db/db mice exhibited significant increase of gene expression in RANKL-RANK signaling, which was essential for osteoclast formation, activation and survival^62, 63^. However, we found no obvious alteration in skeletal RANKL or RANK gene expression after the exposure to PEMF for 12 weeks in the db/db mice, which confirmed the weak effect of PEMF on osteoclast activity and bone resorption. Taken together, we conclude that the anabolic action of PEMF in db/db mice might be associated with the activation of canonical Wnt/β-catenin signaling.

In conclusion, the current study demonstrates that 12 weeks of PEMF stimulation significantly mitigated the deterioration of cancellous bone microarchitecture and decrease of cortical bone thickness in db/db mice. PEMF also significantly improved biomechanical whole-bone structural properties and tissue-level trabecular bone material properties in db/db mice. Moreover, our serum biochemical, bone histomorphometric and qRT-PCR results reveal significant increase of bone formation but not bone resorption in db/db mice after exposure to PEMF for 12 weeks. PEMF upregulated skeletal gene expression of canonical Wnt/β-catenin signaling but not RANKL-RANK signaling in db/db mice. This study enriches our basic understanding to the osteogenetic activity of PEMF, and implies that PEMF might become a clinically applicable treatment modality for improving bone quality in patients with type 2 diabetes. In the future studies, it will be interesting to investigate the residual effects on bone mass, quality and turnover after treatment withdrawal of PEMF both in animals and patients with type 2 DM.
